# Factor XI and XIa Inhibitors: A State-of-the-Art Review Across Clinical Indications

**DOI:** 10.3390/jcm15197431

**Published:** 2026-09-24

**Authors:** Ermioni Oikonomou, Mark Goldin, Alex C. Spyropoulos

**Affiliations:** 1 Institute of Health System Science, Feinstein Institutes for Medical Research, Northwell Health, Manhasset, NY 11030, USA; eoikonomou@northwell.edu (E.O.); mgoldin@northwell.edu (M.G.); 2 Department of Medicine, Anticoagulation and Clinical Thrombosis Services, Northwell Health at Lenox Hill Hospital, New York, NY 10075, USA; 3 Donald and Barbara Zucker School of Medicine at Hofstra, Northwell, Hempstead, NY 11549, USA; 4 Elmezzi Graduate School of Molecular Medicine, Manhasset, NY 11030, USA

**Keywords:** factor XI, factor XIa, anticoagulation, bleeding risk, venous thromboembolism, thromboprophylaxis, secondary stroke prevention, antisense oligonucleotides, monoclonal antibodies, small-molecule inhibitors

## Abstract

Balancing thrombotic and bleeding risk remains the central challenge of anticoagulation, and clinically significant bleeding continues to limit even direct oral anticoagulants, especially in high-bleeding-risk patients. Factor XI (FXI) and activated FXI (FXIa) inhibitors are a mechanistically novel class that seeks to uncouple thrombosis from hemostasis; FXI amplifies clot formation through the intrinsic pathway but contributes little to physiologic hemostasis. This state-of-the-art review examines three classes of FXI/FXIa inhibitors (antisense oligonucleotides, monoclonal antibodies, and small-molecule inhibitors) that are in advanced phase II and III clinical trials, and synthesizes currently available evidence across six indications, including primary thromboprophylaxis in high-bleed-risk orthopedic surgery, primary thromboprophylaxis in end-stage renal disease, stroke prevention in atrial fibrillation, acute coronary syndromes, secondary stroke prevention, and treatment of cancer-associated venous thromboembolism. A pattern emerges, where FXI/FXIa inhibition offers a favorable safety profile across multiple settings and high-risk subgroups, yet clear efficacy has so far been established only in secondary prevention of noncardioembolic ischemic stroke over existing antiplatelet strategies. Although unlikely to completely replace current anticoagulants, these agents may offer important therapeutic options for selected high-risk populations. Ongoing phase II and III trials will further define their potential advantages in areas of unmet clinical need.

## 1. Introduction

The management of patients at risk of or with established thromboembolic disease remains a significant clinical challenge, necessitating tailored anticoagulation strategies that address each patient’s unique risk profile [1,2]. Balancing patient- and disease-related thrombotic and bleeding risk factors is inherently complex, and a growing elderly population with complex comorbidities can make risk stratification even more difficult [2,3,4]. Personalized anticoagulation approaches therefore aim to maintain a balance between preventing thrombosis and avoiding bleeding complications [3,4].

The anticoagulation landscape has evolved substantially, progressing from multi-target agents—unfractionated heparin, low-molecular-weight heparins (LMWHs), and vitamin K antagonists—toward increasingly selective factor inhibition. Approval of the pentasaccharide fondaparinux in 2001 represented a new class of selective and synthetically produced indirect FXa inhibitor anticoagulants that did not have antithrombin activity, with a predictable pharmacokinetic profile that allowed for fixed, non-weight adjusted dosing without routine monitoring [5]. This paved the way for the drug discovery of direct oral anticoagulants (DOACs) that selectively targeted thrombin or factor Xa and were first approved in 2008 [4]. Landmark trials established DOACs as at least as efficacious as established anticoagulants such as low-molecular-weight heparin and warfarin across multiple indications that were generally safer (with less major bleeding and importantly less risk of intracranial hemorrhage) with the added advantages of more predictable pharmacokinetics that allowed for fixed, non-weight-based dosing, and few drug–drug interactions without the need for routine coagulation monitoring [6,7].

Despite this progress, clinically significant bleeding with DOACs persists at appreciable rates—approximately 2–4% per year for major bleeding (including an up to 0.5% rate for intracranial hemorrhage and an up to 2% rate of gastrointestinal bleeding) and up to 10% per year for clinically relevant non-major bleeding [8,9,10]. Importantly, these risks are amplified in high-risk populations such as in patients with advanced chronic kidney disease or active malignancy [11,12,13].

In the past decade or so, new Factor XI (FXI)- or Factor XIa (FXIa)-inhibiting agents have been developed to address the need for safer anticoagulation by uncoupling the mechanisms of thrombosis from hemostasis [14,15,16]. FXI sits within the intrinsic pathway of the coagulation cascade, where it is activated to FXIa by Factor XIIa and thrombin through a positive feedback loop that amplifies and sustains clot formation [14,16,17]. At the same time, FXI appears to play only a minor role in the initial hemostatic response to vessel injury, which is predominantly driven by tissue factor and the extrinsic pathway [14,16,17]. This mechanistic distinction suggests that FXI is preferentially engaged under pathological and prothrombotic conditions such as hyperinflammatory states, proliferation of neutrophil extracellular traps, or placement of medical devices rather than during physiologic hemostasis [14,16,17]. Clinical observations in humans support this notion as well, with congenital FXI deficiency (hemophilia C) being associated with a decreased incidence of cardiovascular events and venous thromboembolism (VTE) without an increase in spontaneous or major (including intracerebral) hemorrhage [18,19]. Similar clinical observations were made in Ashkenazi Jewish populations with severe FXI deficiency [20].

Several mechanistically distinct approaches inducing functional FXI deficiency have been developed [21,22]. Antisense oligonucleotides (ASOs) suppress hepatic FXI synthesis via targeted mRNA degradation; monoclonal antibodies (Mabs) bind and neutralize circulating FXI, FXIa, or both; and orally available small peptidomimetic molecules bind reversibly to the active site of FXIa to block activity [19,22]. Additional classes at earlier stages of investigation include aptamers (short oligonucleotides engineered to mimic antibody-like binding), natural peptide inhibitors derived from tick saliva and snake venom, and N-acetylgalactosamine (GalNAc)-conjugated small interfering RNAs (siRNAs) that silence FXI gene expression [23,24]. Among these, ASOs, Mabs, and small molecules have progressed into phase II and III clinical trials that have yielded mixed results [22].

This state-of-the-art review summarizes the current clinical development of FXI and FXIa inhibitors. Additionally, we examine the completed and ongoing phase II and III trials that are defining the efficacy and safety of these agents across different clinical indications of arterial and venous thromboembolic disease.

## 2. Methods

A literature search was performed in PubMed/MEDLINE, Embase, and the Cochrane Central Register of Controlled Trials through September 2026 using the terms “factor XI inhibitor”, “factor XIa inhibitor,” and individual agent names (asundexian, milvexian, abelacimab, osocimab, fesomersen, IONIS-FXIRx, gruticibart) combined with trial design filters and indication-specific terms. The ClinicalTrials.gov and EU Clinical Trials Register databases were searched to identify ongoing, completed, and terminated trials. Conference proceedings from major cardiovascular and thrombosis meetings (American Heart Association [AHA], American College of Cardiology [ACC], European Society of Cardiology [ESC], and International Society on Thrombosis and Haemostasis [ISTH]; 2020–2026) were reviewed for late-breaking data not yet available in peer-reviewed form. Phase II and phase III randomized controlled trials evaluating any FXI- or FXIa-directed agent in human participants were included regardless of indication, comparator, or completion status. For each trial, study design, agent, dosing, comparator, sample size, follow-up, and primary efficacy and safety endpoints were extracted. During the preparation of this manuscript, the authors used Claude (Opus 5.4; Anthropic, San Francisco, CA, USA) to assist with text editing and drafting the figure. The authors reviewed and edited all output and take full responsibility for the content of the publication.

## 3. Results

The key mechanisms of action and pharmacokinetic/pharmacodynamic (PK/PD) properties of FXI/FXIa inhibitor categories undergoing advanced clinical trials are shown in Table 1.

The results of the phase II and phase III clinical trials of FXI and FXIa inhibitors are summarized in Table 2 and Table 3, respectively. Phase II trials primarily evaluated dose-ranging safety and anticoagulant pharmacokinetic and pharmacodynamic properties, while phase III trials were powered to evaluate clinical efficacy and safety endpoints. Fourteen phase II trials and eight phase III trials have been conducted across six clinical domains: VTE prophylaxis after total knee arthroplasty, thromboprophylaxis in patients with end-stage renal disease (ESRD) requiring hemodialysis, stroke prevention in atrial fibrillation (AF), acute coronary syndrome (ACS), secondary stroke prevention, and cancer-associated VTE. The following results are organized by clinical indication, integrating evidence from both phase II and phase III trials.

### 3.1. Thromboprophylaxis After Total Knee Arthroplasty

Five phase II trials have evaluated FXI/XIa inhibitors for VTE prophylaxis in patients undergoing elective total knee arthroplasty (TKA) (Table 2). The ANT-005 trial randomized 412 TKA patients to a single postoperative intravenous dose of the Mab abelacimab (30 mg, 75 mg, or 150 mg) versus the LMWH enoxaparin 40 mg daily. Overall VTE occurred in 13% (30 mg), 5% (75 mg), and 4% (150 mg) of abelacimab-treated patients compared with 22% in the enoxaparin group, while the 75 mg and 150 mg doses demonstrated superiority over enoxaparin (risk difference: −16.8 percentage points [95% confidence interval (CI), −26.0 to −7.6; *p* < 0.001] and −17.8 percentage points [95% CI, −26.7 to −8.8; *p* < 0.001], respectively). At the same time, major bleeding rates remained at or below 2% across all doses [25]. Similarly, the FOXTROT trial, including 813 TKA patients, showed that another Mab osocimab given preoperatively at 1.8 mg/kg was superior to enoxaparin (total VTE 11.3% vs. 26.3%; p = 0.007), with bleeding rates of 0–4.7% across osocimab groups versus 5.9% for enoxaparin up to postoperative days 10 to 13. All bleeding events consisted of surgical-site bleeding, with no intracranial hemorrhage or bleeding into critical sites [26]. The AXIOMATIC-TKR trial, which randomized 1242 TKA patients to one of seven dose regimens for the small-molecule milvexian (25–200 mg once or twice daily) versus open-label enoxaparin 40 mg daily, demonstrated dose-dependent reduction in VTE (total VTE rates of 21%, 11%, 9%, and 8% for 25 mg, 50 mg, 100 mg, and 200 mg twice daily, respectively, vs. 21% for enoxaparin; *p* < 0.001 for trend). The rates of any bleeding (4% vs. 4%) and major/clinically relevant non-major bleeding (CRNMB) events (1% vs. 2%) were similar between the two groups [27]. In a different open-label phase II clinical trial that included 300 TKA patients, a FXI-ASO initiated 36 days before surgery significantly reduced postoperative VTE compared with enoxaparin. The 300 mg dose was superior for total VTE prevention (4% vs. 30%; *p* < 0.001) and was associated with fewer major or clinically relevant non-major bleeding events (3% vs. 8%) [28]. Two other phase II trials evaluated novel Mabs targeting distinct domains of FXI. Among the two novel Mabs, REGN7508Cat (catalytic domain binding) demonstrated superiority over enoxaparin in the ROXI-VTE-II trial (VTE rates 7% vs. 17%) in 179 TKA patients, whereas REGN9933A2 (selective FXIIa-mediated activation blockade) did not achieve superiority in the ROXI-VTE-I trial (total VTE rates 17% vs. 22%), which included 373 TKA patients. No major or CRNMB events occurred in either ROXI trial [29].

### 3.2. Thromboprophylaxis in End-Stage Kidney Disease on Hemodialysis

Three phase II trials have evaluated FXI/FXIa inhibitors for thromboprophylaxis of patients with end-stage renal disease (ESRD) on hemodialysis, a population facing simultaneously high thrombotic and bleeding risks (Table 2). The Factor XI ASO IONIS-FXIRx was evaluated at 200 mg or 300 mg subcutaneously weekly for 12 weeks versus placebo in 49 patients with ESRD on hemodialysis. Treatment resulted in dose-dependent FXI activity reductions of 56% (200 mg) and 71% (300 mg) versus 4% for placebo, with major bleeding rates of 0% and 6.7% versus 7.7% for placebo [30]. Another phase II trial, the CONVERT trial, evaluated osocimab in 704 patients with ESRD on hemodialysis and found no increase in clinically relevant bleeding (6.9% and 4.9% vs. 7.8% for placebo). However, the composite adverse event endpoint was numerically higher with osocimab (51% and 47% vs. 43%), though not statistically significant [31]. In a similar design, the RE-THINC trial randomized 308 patients with kidney failure on hemodialysis to fesomersen (a GalNAc-conjugated antisense oligonucleotide) at 40 mg, 80 mg, or 120 mg subcutaneously monthly versus placebo for up to 12 months. A dose-dependent and steady state reduction in Factor XI activity was achieved by fesomersen (53.6–86.0% across doses vs. 1.9% for placebo), without increasing major bleeding or CRNMB (event rates of 3.9–6.5% across doses vs. 4.0% for placebo; *p* = 0.78). Interestingly, lower predicted FXI levels were associated with significant reductions in hemodialysis circuit clotting (*p* = 0.002) and arteriovenous access thrombosis (*p* = 0.014), suggesting a potential clinical benefit specific to this population. No phase III trials have been conducted at this point [32].

### 3.3. Stroke Prevention in Atrial Fibrillation

Factor XI/XIa inhibitors have been extensively studied in stroke prevention for atrial fibrillation (AF), including two phase II and three phase III trials (Table 2 and Table 3). The small-molecule asundexian was evaluated in a multicenter, randomized, double-blind study, the PACIFIC-AF trial, with the purpose of dose finding. In total, 755 patients were randomized to asundexian 20 mg or 50 mg once daily versus apixaban 5 mg twice daily over 12 weeks. Asundexian achieved dose-dependent FXIa inhibition reaching 81–90% (20 mg) and 92–94% (50 mg) at trough and peak concentrations, respectively, with significantly lower rates of major/CRNMB compared with apixaban (pooled ratio of incidence proportions 0.33; 90% CI, 0.09–0.97) [33]. Similarly, the AZALEA-TIMI 71 trial randomized 1287 patients with AF to subcutaneous abelacimab 90 mg or 150 mg monthly versus open-label rivaroxaban 20 mg daily (15 mg if creatinine clearance (CrCl) ≤ 50 mL/min). The trial was stopped early by the independent monitoring committee (IDMC) due to a greater-than-anticipated reduction in bleeding with abelacimab, with major or CRNMB reduced by approximately 62–69% across doses. However, despite near-complete FXI suppression (≥97–99%), a potential reduced efficacy signal appeared, with both doses of abelacimab demonstrating numerically higher rates of stroke and systemic embolism compared with rivaroxaban (hazard ratio (HR) 1.65 [90 mg]; HR 1.47 [150 mg]) [34].

The phase III OCEANIC-AF trial provided additional cautionary results. The trial randomized 14,810 patients to asundexian 50 mg versus standard-dose apixaban 5 mg or 2.5 mg twice daily as per label; however, the IDMC terminated the trial early due to a significantly higher incidence of thromboembolic events with asundexian. More specifically, despite a 68% reduction in major bleeding with asundexian, a significant 3.8-fold excess in stroke or systemic embolism with asundexian was observed (1.3% vs. 0.4%; HR 3.79; 95% CI, 2.46–5.83). The net clinical benefit composite outcome significantly favored apixaban (HR 1.61; 95% CI, 1.21–2.15) [35].

There are currently two ongoing phase III trials studying Factor XI/XIa inhibitors for AF. The LIBREXIA-AF trial is comparing milvexian 100 mg twice daily vs. apixaban (5 mg or 2.5 mg twice daily per label) with a goal of enrolling approximately 15,500 patients with AF or atrial flutter. The primary efficacy endpoint is stroke or systemic embolism (noninferiority design), while the primary safety endpoint is ISTH major bleeding/CRNMB [36]. The promising results of the phase II AZALEA-TIMI 71 trial encouraged the ongoing phase III LILAC-TIMI 76 trial evaluating abelacimab 150 mg versus placebo for stroke prevention in approximately 1900 high-risk patients with AF who are unsuitable for standard anticoagulation. The primary efficacy endpoint is stroke or systemic embolism, with major bleeding as the primary safety endpoint [37].

### 3.4. Acute Coronary Syndromes

Factor XI/XIa inhibitors have been investigated in one phase II and one phase III trial for acute coronary syndromes (ACS) (Table 2 and Table 3).

A randomized, placebo-controlled, double-blind trial (PACIFIC-AMI) evaluated asundexian at once-daily doses of 10 mg, 20 mg or 50 mg versus placebo, added to dual antiplatelet therapy (DAPT) in 1601 patients with recent acute MI. Despite the PACIFIC-AMI study demonstrating dose-dependent FXIa inhibition with asundexian reaching approximately 91% with the 50 mg dose, the primary safety endpoint (Bleeding Academic Research Consortium [BARC] type 2, 3, or 5 bleeding) showed no significant difference between pooled asundexian and placebo (HR 0.98; 90% CI, 0.71–1.35). The composite of cardiovascular death, myocardial infarction (MI), stroke, or stent thrombosis was similar between groups (HR 1.05; 90% CI, 0.69–1.61); however, the study was not powered for efficacy [38]. The LIBREXIA-ACS trial subsequently randomized 14,194 patients after a recent ACS to milvexian 25 mg twice daily versus placebo in combination with standard antiplatelet therapy. However, the trial was stopped early for futility at a prespecified interim analysis. Over a median follow-up of 12.2 months, milvexian did not reduce the primary efficacy endpoint of cardiovascular death, myocardial infarction, or ischemic stroke (5.4% vs. 5.1%; HR 1.05; 95% CI, 0.91–1.21; *p* = 0.50), with no benefit for any individual component or for all-cause mortality. Additionally, the principal safety endpoint of intracranial or fatal bleeding (BARC type 3c or 5) was not increased (0.3% vs. 0.3%; HR 1.04; 95% CI, 0.58–1.87; *p* = 0.88) [39].

### 3.5. Secondary Stroke Prevention

Two phase II and two phase III trials have evaluated FXI/FXIa inhibitors for secondary stroke prevention (Table 2 and Table 3). The phase II PACIFIC-Stroke trial randomized 1808 patients with recent noncardioembolic ischemic stroke to asundexian 10 mg, 20 mg, or 50 mg once daily versus placebo, added to usual antiplatelet therapy, with a follow-up of 26–52 weeks. The primary efficacy endpoint of covert brain infarcts on magnetic resonance imaging (MRI) plus recurrent ischemic stroke showed no significant difference between any asundexian dose and placebo (19–22% vs. 19%; *p* = 0.80), while the primary safety endpoint of ISTH major/CRNMB was numerically but not significantly higher with asundexian (3–4% vs. 2%) [40]. In a similar design, the phase II AXIOMATIC-SSP trial randomized 2366 patients with acute noncardioembolic ischemic stroke or transient ischemic attack (TIA) to one of five milvexian dose regimens (25–200 mg once or twice daily) versus placebo, added to dual antiplatelet therapy for 21 days followed by aspirin monotherapy up to day 90. No significant dose–response was observed for the primary composite efficacy outcome of ischemic stroke or covert brain infarcts (15.3–16.7% across milvexian doses vs. 16.8% for placebo). Major bleeding (BARC 3/5) was low and similar across groups (~1–2%) [41].

Following the encouraging safety signals observed in phase II studies, the phase III OCEANIC-STROKE trial randomized 12,327 patients within 72 h of a noncardioembolic ischemic stroke or high-risk TIA to asundexian 50 mg once daily or placebo, added to planned antiplatelet therapy. Over a median follow-up of 567 days, asundexian significantly reduced the primary endpoint of ischemic stroke by 26% (6.2% vs. 8.4%; HR 0.74; 95% CI, 0.65–0.84; *p* < 0.001). Similarly, the key secondary composite of cardiovascular death, myocardial infarction or stroke was also significantly reduced (9.2% vs. 11.1%; HR 0.83; 95% CI, 0.74–0.92; *p* < 0.001), as was disabling or fatal stroke (2.1% vs. 3.0%; HR 0.69; 95% CI, 0.55–0.87). Importantly, major bleeding was not increased in the asundexian arm (1.9% vs. 1.7%; HR 1.10; 95% CI, 0.85–1.44; *p* = 0.46) [42]. The currently ongoing phase III trial, LIBREXIA-STROKE, is evaluating milvexian 25 mg twice daily versus placebo, added to antiplatelet therapy, in approximately 15,000 patients with noncardioembolic ischemic stroke or TIA. The primary efficacy endpoint is ischemic stroke, while major bleeding is set as the principal safety endpoint [43].

### 3.6. Cancer-Associated VTE

Two phase III trials have examined abelacimab for the treatment of cancer-associated VTE (Table 3). The ASTER trial compared abelacimab 150 mg subcutaneously monthly with apixaban in approximately 1150 patients with cancer-associated VTE, while a second trial, MAGNOLIA, compared abelacimab 150 mg subcutaneously monthly versus the LMWH dalteparin in approximately 417 patients with gastrointestinal or genitourinary cancers. The primary endpoint was recurrent VTE, with major/CRNMB as the primary safety endpoint for both trials. However, both phase III trials were terminated early; ASTER following an interim analysis indicating inferior efficacy of abelacimab compared with apixaban for the prevention of recurrent VTE, while MAGNOLIA was terminated following a sponsor decision. Full results have not yet been published [44,45,46].

### 3.7. Post Hoc and Subgroup Analyses

Several secondary analyses from the phase II and III trials have provided clinically important insights into the behavior of FXI/FXIa inhibitors in specific clinical contexts. A post hoc analysis by Patel et al. examined periprocedural bleeding among patients undergoing invasive procedures in the AZALEA-TIMI 71 trial [47]. Among the 441 patients who underwent 920 procedures (75.7% low bleeding risk, 74.6% elective), periprocedural major or CRNMB occurred in 1.2% of the procedures in the abelacimab arm versus 2.2% in the rivaroxaban arm (RR 0.54; 95% CI, 0.19–1.58). Abelacimab was continued periprocedurally in 56% of procedures and withheld for a median of 44 days for high-bleed-risk procedures or surgeries, while rivaroxaban was interrupted as per established protocols [47]. A separate analysis from AZALEA-TIMI 71 examined outcomes among patients receiving concomitant antiplatelet therapy at baseline [48]. Al Said et al. found that in the rivaroxaban arm, concomitant antiplatelet therapy increased major/CRNMB from 7.7 to 10.6 per 100 patient-years. In the abelacimab arm, rates were 2.5–3.5 per 100 patient-years with antiplatelet therapy and 2.7–3.1 without [48]. Two additional prespecified analyses of AZALEA-TIMI 71 examined the consistency of abelacimab’s bleeding advantage across key subgroups. In an analysis by kidney function (median creatinine clearance [CrCl] 71 mL/min, with 20.6% of patients having a CrCl ≤ 50 mL/min), abelacimab reduced major or CRNMB relative to rivaroxaban consistently across the spectrum of renal function [49]. A separate analysis by age found a consistent relative reduction in bleeding regardless of age, with a greater absolute benefit in older patients (major or CRNMB reduced by 6.2% in those ≥75 years vs. 4.2% in those <75 years) [50].

Interestingly, in the phase II PACIFIC-Stroke trial, a post hoc analysis examining the data-driven composite endpoint of recurrent symptomatic ischemic stroke and transient ischemic attack suggested that asundexian 50 mg reduced this endpoint versus placebo (HR 0.64; 90% CI, 0.41–0.98). Critically, this benefit appeared to be driven by patients with atherosclerotic stroke subtypes. In the prespecified subgroup of patients with stroke attributed to large-artery atherosclerotic disease by Trial of Org 10172 in Acute Stroke Treatment (TOAST) classification, asundexian 50 mg was associated with a large, non-significant relative risk reduction in recurrent symptomatic ischemic stroke (8% vs. 15%; HR 0.53; 90% CI, 0.24–1.17). Another related imaging analysis found that among patients with evidence of any extracranial or intracranial atherosclerosis supplying the qualifying infarct, asundexian 50 mg was associated with a reduction in recurrent ischemic stroke or TIA (HR 0.39; 90% CI, 0.18–0.85) [40]. A separate MRI-based post hoc analysis further supported this pattern, showing that asundexian 50 mg reduced recurrent ischemic stroke or incident covert infarcts in patients with large, multiple, or cortical infarcts (HR 0.71; 95% CI, 0.45–1.11), but not in those with single small subcortical infarcts (HR 1.14; 95% CI, 0.62–2.10) [51].

## 4. Discussion

Despite substantial advances in anticoagulation, clinically significant bleeding remains a limitation of all currently available anticoagulants, particularly in high-risk populations [52]. Factor XI/XIa inhibitors were developed to address this unmet need by selectively targeting the intrinsic coagulation pathway, aiming to uncouple and thus target pathological thrombosis from physiological hemostasis [14]. The accumulated clinical evidence now allows for a detailed assessment of where these therapeutic agents succeed and where they fall short, with the underlying pathophysiology of each condition of interest emerging as a key determinant of efficacy, in addition to mechanism of action (MOA) and key PK/PD characteristics as well as degree of FXI inhibition as potential contributors.

### 4.1. Venous Thromboembolism Prophylaxis

Factor XI/XIa inhibitors have been evaluated in five phase II trials for VTE prophylaxis after TKA, a clinical setting that has traditionally served as the primary model for the evaluation of novel anticoagulants [19]. The results were highly consistent, demonstrating that mechanistically diverse FXI/XIa inhibitors, including an ASO, two mAbs, a small-molecule FXIa inhibitor and the novel catalytic-domain-binding antibody REGN7508Cat, achieved efficacy comparable or superior to a “gold-standard” anticoagulant such as the LMWH enoxaparin in the setting of primary thromboprophylaxis, while at the same time maintaining similar or improved bleeding profiles [25,26,27,28,29]. Additionally, a clear dose–response pattern emerged across trials, with higher-intensity FXI inhibition producing greater reductions in VTE, underscoring the importance of achieving sufficient FXI activity suppression for clinical efficacy [27,28]. The only notable exception, the ROXI-VTE I trial, provided further mechanistic insights. Because REGN9933A2 selectively blocks FXIIa-mediated FXI activation rather than FXIa catalytic activity directly, its outcome relative to enoxaparin suggests that thrombin-mediated FXI activation via the positive feedback loop may be an important driver of postoperative thrombosis [29]. Overall, these results align with the understanding that postoperative VTE after joint replacement involves contact activation through artificial surfaces and tissue trauma, a process that is substantially FXI-dependent [14,29]. By contrast, the extrinsic pathway’s predominant role in the initial hemostatic response at the surgical wound also explains why these agents largely spared major bleeding [14]. Thus, primary thromboprophylaxis after major surgery (especially in high thrombotic and high-bleed-risk groups such as major orthopedic or abdominal cancer surgery) may represent the most mechanistically favorable indication for Factor XI/XIa inhibition. Despite the strength of the phase II data across multiple agents and mechanistic classes, no phase III trials are being conducted for this indication. Whether the observed superiority will be maintained in larger phase III studies with longer follow-up and in direct comparisons with DOACs remains to be determined.

### 4.2. Thromboprophylaxis of End-Stage Kidney Disease on Hemodialysis

Patients with ESRD on hemodialysis represent a highly challenging anticoagulation population, where conventional anticoagulants are associated with increased bleeding risk, unreliable pharmacokinetics, and often suboptimal efficacy for both systemic thromboembolism and hemodialysis circuit clotting [53,54,55]. Therefore, these patients represent a compelling population for Factor XI/XIa inhibition [53]. Data from the three completed phase II trials have shown consistently that FXI suppression can be achieved safely in this population without increasing major or clinically relevant bleeding relative to a placebo [30,31,32]. Particularly informative are findings from the RE-THINC trial, where lower predicted FXI levels achieved with fesomersen were associated with significant reductions in both hemodialysis circuit clotting and arteriovenous access thrombosis [32]. This observation is reinforced mechanistically, since hemodialysis circuits activate the contact (intrinsic) pathway through the exposure of blood to artificial membrane surfaces, a process driven by FXIIa-mediated FXI activation [56]. However, despite the promising results of phase II trials, the absence of phase III data remains a significant gap in this high-risk population. Adequately powered phase III trials evaluating hard clinical endpoints are needed to establish whether phase II safety and mechanistic signals translate into meaningful clinical benefit.

### 4.3. Stroke Prevention in Atrial Fibrillation

The stroke prevention in AF indication has yielded more cautionary results for Factor XI/XIa inhibitors. While the phase II PACIFIC-AF and AZALEA-TIMI 71 trials demonstrated significant bleeding reductions with asundexian and abelacimab compared with DOACs, the well-powered phase III OCEANIC-AF trial revealed significantly reduced efficacy with asundexian compared to apixaban [33,34,35]. Specifically, a 3.8-fold excess in stroke or systemic embolism with asundexian 50 mg compared with apixaban led to early termination of the OCEANIC-AF trial. Additionally, the net clinical benefit composite of stroke, systemic embolism, or major bleeding significantly favored apixaban [35]. Similar efficacy signals from the AZALEA-TIMI 71 trial, in which both doses of abelacimab were associated with numerically higher stroke and systemic embolism rates compared with rivaroxaban, raise the possibility of a class-level limitation. However, whether this represents a true class effect or is specific to individual agents or dosing strategies remains uncertain and may be clarified by the ongoing phase III LIBREXIA-AF and LILAC-TIMI 76 trials [34]. These findings possibly reflect the fundamental pathophysiology of AF-related thromboembolism—left atrial appendage thrombus formation predominantly driven by blood stasis and tissue factor-mediated thrombin generation through the extrinsic pathway, with FXI playing only a secondary amplification role [57,58]. While direct thrombin and factor Xa inhibitors interrupt these final common coagulation pathways directly with broader suppression of thrombin generation, FXIa inhibition by blocking thrombin amplification alone may be insufficient to prevent cardioembolic events after thrombin activation from stasis-driven mechanisms [35,59]. In addition, the low dose of asundexian (50 mg), which achieved 92–94% inhibition of FXI based on a non-standardized FXI assay, may have contributed to this lack of efficacy [33]. Nevertheless, the same asundexian 50 mg once-daily dose was associated with a reduction in recurrent ischemic stroke in OCEANIC-STROKE, suggesting that this dose provides biologically meaningful FXIa inhibition [42]. While insufficient FXIa inhibition may have contributed to the OCEANIC-AF findings, the pathophysiologic mechanisms discussed above may have played a more substantial role in the observed lack of efficacy. Despite these limitations, the substantial reduction in bleeding observed with FXI/FXIa inhibitors remains clinically meaningful and may still offer a valuable treatment option for patients in whom the bleeding risk associated with conventional anticoagulation outweighs its benefit [60]. Secondary analyses from AZALEA-TIMI 71 confirmed that abelacimab’s bleeding advantage was consistent across clinically relevant subgroups, including patients on concomitant antiplatelet therapy, renal function, and age [48,49,50]. Importantly, despite abelacimab’s long half-life (25–28 days), no excess periprocedural bleeding risk was demonstrated, an important practical consideration for a long-acting anticoagulant. These observations have informed the design of the ongoing phase III LILAC-TIMI 76 trial, which is evaluating abelacimab versus placebo specifically in high-risk AF patients deemed unsuitable for standard anticoagulation [34]. Finally, the phase III LIBREXIA-AF trial comparing milvexian 100 mg twice daily versus apixaban in the broader AF population will clarify whether a different small-molecule FXIa inhibitor at higher doses can achieve noninferiority for stroke prevention compared to a DOAC and retain safety advantages [61].

### 4.4. Acute Coronary Syndromes

The investigation of FXI/XIa inhibitors in ACS has yielded no clear benefit compared to conventional antithrombotic strategies. In the phase II PACIFIC-AMI trial, asundexian added to dual antiplatelet therapy demonstrated neither a meaningful reduction in major bleeding nor a signal of efficacy across any dose, despite achieving reasonable FXIa inhibition (91% FXIa inhibition at the 50 mg dose) [38]. The subsequent phase III LIBREXIA-ACS trial, which compared milvexian 25 mg twice daily with placebo added to standard antiplatelet therapy in patients with a recent ACS, was terminated early after an interim analysis determined futility for the primary efficacy endpoint of cardiovascular death, MI, or ischemic stroke, though without an increase in intracranial or fatal bleeding [39]. These outcomes may in part reflect fundamental differences in the pathobiology of high flow arterial thrombosis in ACS versus the venous and contact pathway-driven thrombosis where FXI inhibitors have shown efficacy [22]. Furthermore, the selection of milvexian 25 mg twice daily for LIBREXIA-ACS, a dose chosen to minimize bleeding risk on top of potent dual antiplatelet therapy, may have provided insufficient FXIa inhibition to meaningfully reduce thrombin generation in this high tissue factor environment [61]. Exploratory analyses may possibly identify subgroups, such as patients with recurrent thrombotic events despite DAPT, in whom FXI inhibition could provide targeted benefit [61]. However, the data do not currently support Factor XI/XIa inhibitors as a viable option for the ACS population.

### 4.5. Secondary Stroke Prevention

Secondary prevention of noncardioembolic ischemic stroke has emerged as the most promising clinical application and potential first drug approval for FXI/FXIa inhibitors [42]. The phase II PACIFIC-Stroke and AXIOMATIC-SSP trials comparing the small-molecule FXIa inhibitors asundexian and milvexian in addition to background antiplatelet therapy versus placebo for secondary stroke prevention did not meet their primary composite endpoints, though this may in part reflect the inclusion of covert brain infarcts on MRI (a surrogate of uncertain clinical significance) that likely masked a true treatment signal [40,41]. Nevertheless, both studies produced encouraging exploratory findings. Exploratory post hoc analysis of these trials consistently demonstrated a reduction in recurrent symptomatic ischemic stroke and TIA compared with placebo, with the benefit appearing to be driven primarily by atherosclerotic stroke mechanisms and by infarct patterns typical of atheroembolism [40,51]. The phase III OCEANIC-STROKE trial validated these exploratory signals in patients with noncardioembolic ischemic stroke or high-risk TIA, demonstrating a significant reduction in ischemic stroke with asundexian 50 mg added to antiplatelet therapy versus antiplatelet therapy alone [42]. Subgroup analyses showed that the benefit was generally consistent across key subgroups, including patients with large-artery atherosclerosis and those with embolic stroke of undetermined source (ESUS) [42]. The ESUS subgroup finding is particularly noteworthy, as all prior phase III DOAC trials in ESUS failed to demonstrate superiority over aspirin for recurrent stroke prevention in addition to increased bleeding [62,63]. The mechanistic basis for efficacy in noncardioembolic stroke potentially lies in the predominance of atherothrombotic mechanisms, where plaque-driven thrombin generation subsequently activates FXI-mediated amplification [14,64]. Unlike other cardiovascular settings, where adding an anticoagulant to antiplatelet therapy has consistently increased major bleeding, FXIa inhibition was not associated with excess bleeding [42]. These findings suggest that the selective inhibition of pathological, rather than physiological, coagulation may enable safe dual antithrombotic regimens in this context [14,22]. Asundexian is presently undergoing review by the Food and Drug Administration in the US for this indication. Finally, the ongoing phase III LIBREXIA-STROKE trial evaluating milvexian 25 mg twice daily in a similar population will determine whether the benefit observed with asundexian represents a class effect of small-molecule FXIa inhibitors [61]. A consistent benefit of FXI inhibition could represent a major advance in secondary stroke prevention and influence future standards of care.

### 4.6. Cancer-Associated VTE

Cancer-associated VTE is among the highest-risk thrombotic conditions encountered in clinical practice, driven by tumor-mediated hypercoagulability, endothelial disruption and systemic inflammation [65]. At the same time the bleeding risks of conventional anticoagulants are amplified by tumor-related factors, thrombocytopenia, and drug–drug interactions with anticancer therapies [65,66]. The rationale for FXI inhibition in this setting is supported by the pathophysiology of cancer-associated hypercoagulability. Specifically, tissue factor expression by tumor cells, inflammatory cytokine-driven coagulation activation, and neutrophil extracellular trap formation all contribute to a high degree of thrombin generation through FXI-mediated amplification, making FXI an attractive therapeutic target [67,68]. Two phase III trials—ASTER (abelacimab versus apixaban) and MAGNOLIA (abelacimab versus dalteparin)—were designed to test whether abelacimab could provide effective VTE treatment with less bleeding in this vulnerable population. However, both trials were terminated early and full results have not yet been published [44,45]. The premature termination of the ASTER trial raises important questions about whether FXI inhibition provides sufficient antithrombotic efficacy for the treatment (as opposed to prevention) of established VTE, where the thrombus burden is already substantial and may require more potent final common pathway inhibition utilizing both intrinsic and extrinsic pathway mechanisms for resolution. The cancer-associated VTE indication remains an important but unresolved question for this class of agents, with more data needed to establish whether FXI/FXIa inhibition can maintain adequate efficacy while meaningfully reducing bleeding complications.

### 4.7. Off-Target Effects and Reversal Strategies

Beyond bleeding, attention has turned to potential off-target effects of FXI inhibition tied to its role outside of coagulation. Experimental data suggest liver-derived FXI is cardioprotective in HFpEF, where its proteolytic activity cleaves and activates BMP7 to engage the BMP–SMAD1/5 pathway, limiting cardiac fibrosis and inflammation [69]. This raises an interesting question as to whether sustained FXI suppression could diminish this protection and, in vulnerable patients, accelerate HFpEF or adverse remodeling [69]. The impact of FXI inhibition on postoperative wound healing or swelling remains speculative and largely preclinical, although data from phase II clinical trials suggests that in tissues of low fibrinolytic activity (such as orthopedic surgery) suppression of FXI does not compromise surgical wound closure or hemostasis [70]. The bleeding phenotype of FXI deficiency is tissue-dependent and pronounced in fibrinolytic-rich tissues (oral mucosa, nasopharynx, genitourinary tract) but negligible in low-fibrinolytic sites, where even severely deficient patients (<20% activity) achieve uncomplicated hemostasis in abdominal and orthopedic surgery [70].

No specific antidote is currently being studied for any FXI/FXIa inhibitor, given the premise of this class of anticoagulants that hemostasis is minimally affected and the overall excellent safety profile seen from phase II and III clinical trials. Reversal strategies remain non-targeted and are based on each agent’s mechanism of action and pharmacokinetic profile [52,71]. For the ASOs and siRNA agents, which lower FXI synthesis, hemostasis can be restored by repleting FXI with fresh frozen plasma or FXI concentrates. This approach, however, fails for Mabs, since infused FXI is simply neutralized by circulating antibody. For Mabs, only non-specific bypassing agents (rFVIIa or PCC) have potential to restore hemostasis, but their long half-life plus high plasma-protein binding may make them difficult to reverse. Across these agents, tranexamic acid is a valuable adjunctive option [71].

## 5. Conclusions

Factor XI/XIa inhibitors represent a mechanistically novel class of anticoagulants that aim to uncouple thrombosis from hemostasis by selectively targeting the intrinsic coagulation pathway [14,16]. The clinical evidence to date based on data from advanced clinical trials reveals an emerging pattern (Figure 1): while this class of anticoagulants shows an overall improved safety profile compared to existing antithrombotic strategies across multiple indications, clear efficacy has only been shown with asundexian in secondary stroke prevention (including ESUS), where dual pathway inhibition represents a potential new “standard-of-care” for this indication pending confirmation of a class effect in the ongoing LIBREXIA-STROKE trial, while superior efficacy compared to a “gold standard” anticoagulant such as LMWH has been suggested across multiple agents in primary thromboprophylaxis of high-bleed-risk orthopedic surgery.

Safety advantages of this class of anticoagulants has been seen in key high-risk patient subgroups, including patients with ESRD and advanced age, those on background antiplatelet therapies, or as simplified uninterrupted strategies in patients on chronic anticoagulants requiring low-to-moderate bleed risk elective surgical interventions. Additionally, the class has been well tolerated, with no serious off-target adverse effects observed to date, although the theoretical loss of FXI’s cardioprotective and other pleiotropic functions warrants long-term surveillance. Its principal practical limitation is the absence of a specific antidote, as reversal currently relies on non-targeted strategies (tranexamic acid, FFP, PCC, or rFVIIa) with FXI replacement effective only for ASOs. Efficacy, however, has not been established across multiple indications, with emerging phase III clinical trial data failing to demonstrate efficacy in ACS, stroke prevention in AF, and cancer-associated VTE. Whether this lack of efficacy in certain clinical situations is based on incomplete suppression of thrombin generation via intrinsic pathway mechanisms alone compared to more robust suppression of thrombin generation with anticoagulants that target the final common pathway (i.e., DOACs), the need for greater (i.e., >99%) suppression of FXIa activity, or agent-specific MOA or PK/PD considerations, remains to be seen. The LIBREXIA-STROKE trial that is studying milvexian will determine whether the benefit observed with asundexian in OCEANIC-STROKE is a class effect, while the LIBREXIA-AF (milvexian vs. apixaban) and LILAC trials (abelacimab vs. placebo) will assess whether FXIa inhibition with either a small molecule or mAb FXI inhibition can provide stroke prevention comparable to DOACs in AF with a better safety profile or in high-risk AF patients with high bleed risk unsuitable for anticoagulants [43,72]. FXI/FXIa inhibitors as a class are unlikely to replace conventional anticoagulants across all indications but instead may represent a targeted intervention for specific clinical settings of high thrombotic or high bleed risk, where pathological thrombus formation is predominantly driven by FXI-mediated amplification, and where potentially safer anticoagulation can translate into net clinical benefit [25,42]. This includes ESRD, noncardioembolic ischemic stroke, primary thromboprophylaxis of patients with cancer, stroke prevention of AF in high-risk patients, or in the treatment of high-bleed-risk patients with VTE. In addition, FXI/FXIa inhibitors with MOAs that include relatively rapid onset but slower offset of anticoagulant activity (such as mAbs) may be ideal agents to use in hospital settings that require extended thromboprophylaxis post-discharge (such as orthopedic/abdominal cancer surgery or for acute medically ill patients). Further phase II trials across multiple indications and agent classes as well as ongoing phase III trials will determine whether this class of anticoagulants will be able to provide clear advantages in areas of unmet clinical need.

## Figures and Tables

**Figure 1:**
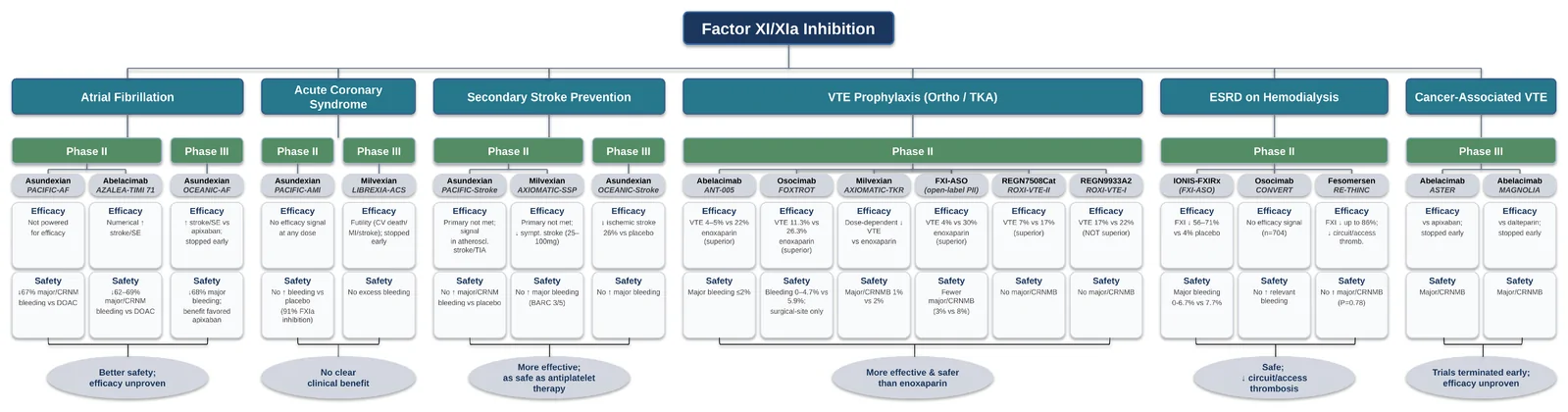
The landscape of Factor XI/XIa inhibitor clinical trials across six major therapeutic indications. A summary of phase II and III clinical trials evaluating Factor XI/XIa inhibition in atrial fibrillation, acute coronary syndrome, secondary stroke prevention, venous thromboembolism prophylaxis following total knee arthroplasty, end-stage renal disease requiring hemodialysis, and cancer-associated venous thromboembolism. Key efficacy and safety findings and the overall clinical implications for each indication are presented. The arrows indicate the direction of effect (↑ increase, ↓ decrease).

**Table 1:** Mechanism of action and pharmacokinetics of common strategies for Factor XI/XIa inhibitors.

Agent	Small Peptidomimetic Molecules	Antisense Oligonucleotides (ASO)	Monoclonal Antibodies (mAb)
**Medication name**	Asundexian, Milvexian, ONO-7684	ISIS-FXIRx, fesomersen (IONIS-FXI-LRx/LICA)	Abelacimab, Xisomab, Osocimab, MK-2060
**Mechanism of action**	Direct inhibition of FXIa	Inhibition of FXI biosynthesis	Direct inhibition of FXI/FXIa
**Route of administration**	IV or oral	SC	IV or SC
**Dosing**	Daily	Weekly	Monthly
**Onset of action**	Minutes/Hours	Weeks	Hours
**Offset of action**	Minutes/Hours	Weeks	Weeks
**Half-life (t½)**	11–21 h	10–20 d	20–44 d
**Renal excretion**	Yes	No	No
**Hepatic metabolism**	CYP3A4 or P-gp	No	No

Abbreviations: ASO: antisense oligonucleotide; CYP: cytochrome P450; d: days; FXI: Factor XI; FXIa: activated Factor XI; h: hours; IV: intravenous; LICA: ligand-conjugated antisense; mAb: monoclonal antibody; P-gp: P-glycoprotein; SC: subcutaneous; t½: half-life.

**Table 2:** Design and outcomes of phase II trials of Factor XI/FXIa inhibitors.

Study Name	Medication Name	Type of Drug	Dose	Comparator	Condition of Interest	N	Status	Duration—Follow-Up	Results/Endpoints	Secondary Endpoints
**ANT-005 TKA** (EudraCT 2019-003756-37)	Abelacimab	Monoclonal antibody	• **Post-op single dose:** - 30 mg IV - 75 mg IV - 150 mg IV	Enoxaparin 40 mg SC QD	VTE prophylaxis (TKA)	412	Completed	• Day 8–12 venography; • Follow-up to day 30 • Extended follow-up to day 110	• **75 mg/150 mg:** superior to enoxaparin (5% 4% vs. 22% VTE; *p* < 0.001) **• 30 mg:** noninferior to enoxaparin (13% vs. 22%; *p* = 0.08 for superiority) **• Major/CRNMB:** 2%, 2%, 0% vs. 0%	• Extent of thrombosis on venography; • Clinically relevant bleeding through day 30 and day 110
**FOXTROT** (NCT03276143)	Osocimab	Monoclonal antibody	• **Post-op:** - 0.3 mg/kg IV - 0.6 mg/kg IV - 1.2 mg/kg IV - 1.8 mg/kg IV • **Pre-op:** - 0.3 mg/kg IV - 1.8 mg/kg IV	• Enoxaparin 40 mg SC QD or, • Apixaban 2.4 mg BID (exploratory)	VTE prophylaxis (TKA)	813	Completed	• Day 10–13 venography; • Follow-up to day 150 post-op	• **Preop:** 1.8 mg/kg superior to enoxaparin (11.3% vs. 26.3%; *p* = 0.007) • **Post-op:** 0.6–1.8 mg/kg: Noninferior to enoxaparin (15.7–17.9% vs. 26.3%) • No increase in major/CRNMB [0–4.7% (osocimab) vs. 5.9% (enoxaparin) vs. 2% (apixaban)]	• **Secondary efficacy**: VTE composite through day 150 • **Secondary safety:** clinically relevant bleeding through day 150
**AXIOMATIC-TKR ** (NCT03891524)	Milvexian	Small molecule	**• BID**: - 25 mg - 50 mg - 100 mg - 200 mg **or**, • **QD:** - 25 mg - 50 mg - 200 mg	Enoxaparin 40 mg SC QD	VTE prophylaxis (TKA)	1242	Completed	• Day 10–14 venography; • Follow-up to day 30	• **Dose-dependent VTE reduction**: - BID: 21%, 11%, 9%, 8% vs. 21% enoxaparin; *p* < 0.001 for trend • **Any bleeding**: 4% vs. 4%; • **Major/CRNMB**: 1% vs. 2%	• Symptomatic VTE, PE or death through day 30 • Extent of thrombosis on venography
**FXI-ASO TKA** (NCT01713361)	FXI-ASO	Antisense oligonucleotide	• **36 days pre-op:** - 200 mg SC - 300 mg SC (multiple dose regimen)	Enoxaparin 40 mg SC QD	VTE prophylaxis (TKA)	300	Completed	• Day 8–12 venography • Follow-up to 3 months post-op	**• 200 mg**: noninferior to enoxaparin (27% vs. 30%; *p* = 0.57) • **300 mg**: superior to enoxaparin (4% vs. 30% VTE; *p* < 0.001) • **Major/CRNMB**: 3% (200 mg) vs. 3% (300 mg) vs. 8% (enoxaparin)	• Symptomatic VTE, PE or death through follow-up • Extent of thrombosis on venography
**ROXI-VTE-I** (NCT05618808)	REGN9933A2	Anti-FXI mAb, apple 2 domain	• **Post-op single dose:** 300 mg IV	• Enoxaparin 40 mg SC QD or, • Apixaban 5 mg BID (exploratory)	VTE prophylaxis (TKA)	373	Completed	• Day 10 (±2) venography; • Median follow-up 74 days	• Not superior to enoxaparin (17% vs. 22%; posterior probability 78.5%) • **Major/CRNMB**: 0% vs. 0%	• Symptomatic VTE, PE or death through follow-up • DVT incidence on venography • Treatment-emergent adverse events
**ROXI-VTE-II** (NCT06454630)	REGN7508Cat	Anti-FXI mAb, catalytic domain	• **Post-op single dose:** 250 mg IV	Enoxaparin 40 mg SC QD	VTE prophylaxis (TKA)	179	Completed	• Day 10 (±2) venography; • Median follow-up 74 days	• Superior to enoxaparin (7% vs. 17%; posterior probability 99.8%) • **Major/CRNMB**: 0% vs. 0%	• Symptomatic VTE, PE or death through follow-up • DVT incidence on venography • Treatment-emergent adverse events
**FXI-ASO ESRD** (NCT02553889)	IONIS-FXIRx	Antisense oligonucleotide (2nd generation)	• 200 mg SC • 300 mg SC (weekly regimen for 12 weeks)	Placebo	ESRD on hemodialysis	49	Completed	• Follow-up to day 162	• **FXI activity reduction** (Day 85; dose-dependent): - 56.0% (200 mg), - 70.7% (300 mg) - 3.9% (placebo) •**Pharmacodynamics**: - Reduced FXI antigen - Dose-dependent aPTT prolongation • **Major bleeding:** - 0% (200 mg), - 6.7% (300 mg), - 7.7% placebo	• **PK/PD parameters:** - FXI antigen - aPTT - INR - Safety/tolerability
**CONVERT** (NCT04523220)	Osocimab	Anti-FXIa monoclonal antibody	• 105 mg SC loading → 52.5 mg monthly • 210 mg SC loading → 105 mg monthly	Placebo	ESRD on hemodialysis	704	Completed	• Treatment ≤ 18 months (minimum 6 months)	**• Clinically relevant bleeding:** - 6.9% (low dose) - 4.9% (high dose) - 7.8% (placebo) • **Adverse events (composite):** - 51% (low dose) - 47% (high dose) - 43% (placebo)	• **PK/PD parameters: -** Medication plasma levels - FXIa inhibition - aPTT
**RE-THINc ESRD ** (NCT04534114)	Fesomersen (GalNAc-ASO)	Antisense oligonucleotide (2nd generation)	• 40 mg SC • 80 mg SC • 120 mg SC (monthly)	Placebo	ESRD on hemodialysis	308	Completed	• Follow-up to 12 months	• **FXI activity reduction** (Dose-dependent; steady state): - 53.6% (40 mg) - 71.3% (80 mg) - 86.0% (120 mg) - 1.9% (placebo) • **Major/CRNMB** (*p* = 0.78): - 6.5% (40 mg) - 5.1% (80 mg) - 3.9% (120 mg) - 4.0% (placebo) • Reduced HD circuit clotting (*p* = 0.002) • Reduced AV-access thrombosis (*p* = 0.014)	• Major atherothrombotic events • AV-access bleeding • PK/PD (FXI levels, aPTT) • Safety
**PACIFIC-AF** (NCT04218266)	Asundexian	Small molecule	• 20 mg QD • 50 mg QD	Apixaban 5 mg BID	Atrial Fibrillation	755	Completed	12 weeks	**• FXIa inhibition**: - 81–90% (20 mg) - 92–94% (50 mg) • **Bleeding (ISTH major/CRNMB):** RR 0.33 vs. apixaban (90% CI 0.09–0.97)	**•** Any bleeding **•** ISTH major bleeding/CRNMB **•** Adverse events; **•** Exploratory thrombotic events (ischaemic stroke, MI, CV death, systemic embolism)
**AZALEA-TIMI 71** (NCT04755283)	Abelacimab	Anti-FXI monoclonal antibody	• 90 mg SC • 150 mg SC (monthly)	Rivaroxaban 20 mg QD (15 mg if CrCl ≤ 50)	Atrial Fibrillation	1287	Completed (stopped early by IDMC for benefit)	• Median follow-up 2.1 years	• **FXI suppression**: ~97% (90 mg), ~99% (150 mg) • **Bleeding (ISTH major/CRNMB):** - HR 0.31 (90 mg) (95% CI 0.19–0.51) - HR 0.38 (150 mg) (95% CI 0.24–0.60) vs. rivaroxaban (*p* < 0.001)	• **Major bleeding:** - HR 0.26 (90 mg) (95% CI 0.12–0.57) - HR 0.33 (150 mg) (95% CI 0.16–0.66) • **Any bleeding:** - HR 0.46 (90 mg) (95% CI 0.33–0.64) - HR 0.68 (150 mg) (95% CI 0.51–0.91) • **Stroke/systemic embolism:** - HR 1.65 (90 mg) (95% CI 0.64–4.25) - HR 1.47 (150 mg) (95% CI 0.56–3.85) • **Net clinical outcome:** - HR 0.58 (90 mg) (95% CI 0.41–0.81) - HR 0.55 (150 mg) (95% CI 0.39–0.77))
**PACIFIC-AMI** (NCT04304534)	Asundexian	Small molecule	• 10 mg QD • 20 mg QD • 50 mg QD	Placebo (+DAPT)	Recent acute MI	1601	Completed	• Follow-up to 6–12 months (median 368 days)	• **FXIa inhibition (week 4; dose-dependent):** ~65% (10 mg) ~79% (20 mg) ~91% (50 mg) • **Bleeding (BARC type 2/3/5**): - 7.6% (10 mg), - 8.1% (20 mg), - 10.5% (50 mg) - 9.0% (placebo) (HR 0.98 (90% CI 0.71–1.35) • No difference in CV death, MI, stroke, or stent thrombosis [HR 1.05 (90% CI 0.69–1.61)]	• **Any bleeding: ** HR 0.90 (90% CI 0.73–1.11) • **CV death/MI/stroke/stent thrombosis:** HR 1.05 (90% CI 0.69–1.61)
**PACIFIC-STROKE** (NCT04304508)	Asundexian	Small molecule	• 10 mg QD • 20 mg QD • 50 mg QD	Placebo (+antiplatelet therapy)	Noncardioembolic ischemic stroke	1808	Completed	• Follow-up to 26–52 weeks (primary analysis at 26 weeks)	• **Primary efficacy** (composite of covert brain infarcts + recurrent ischemic stroke): - 19% (10 mg) - 22% (20 mg) - 20% (50 mg) - 19% (placebo) (*p* = 0.80) • **Bleeding (ISTH major/CRNMB**: - 4% (10 mg) - 3% (20 mg) - 4% (50 mg) - 2% (placebo) (HR 1.57 (90% CI 0.91–2.71)	• **Recurrent ischemic stroke; any stroke; CV death/MI/stroke; all bleeding:** Not statistically significant • **Post hoc: ** stroke/TIA reduced with 50 mg (HR 0.64; 90% CI 0.41–0.98)
**AXIOMATIC-SSP** (NCT03766581)	Milvexian	Small molecule	**• QD:** - 25 mg • **BID**: - 25 mg - 50 mg - 100 mg - 200 mg	**•** Placebo + DAPT (×21 days) **•** Placebo + Aspirin (up to day 90)	Acute noncardioembolic ischemic stroke/TIA	2366	Completed	90 days	• **Primary efficacy** (ischemic stroke + covert brain infarct)**:** No significant dose–response [16.8% (placebo) vs. 15.3–16.7% (milvexian)] • **Major bleeding (BARC 3/5):** No increase (~1–2% across groups)	• **Symptomatic ischemic stroke:** Numerically lower at doses 25–100 mg BID (~30% RRR vs. placebo)

Abbreviations: AF: atrial fibrillation; AMI: acute myocardial infarction; aPTT: activated partial thromboplastin time; ASO: antisense oligonucleotide; AV: arteriovenous; BARC: Bleeding Academic Research Consortium; BID: twice daily; CI: confidence interval; CrCl: creatinine clearance; CRNMB: clinically relevant non-major bleeding; CV: cardiovascular; DAPT: dual antiplatelet therapy; DVT: deep vein thrombosis; ESRD: end-stage renal disease; FXI: Factor XI; FXIa: activated Factor XI; GalNAc: N-acetylgalactosamine; HD: hemodialysis; HR: hazard ratio; IDMC: independent data monitoring committee; INR: international normalized ratio; ISTH: International Society on Thrombosis and Haemostasis; IV: intravenous; mAb: monoclonal antibody; MI: myocardial infarction; PD: pharmacodynamics; PE: pulmonary embolism; PK: pharmacokinetics; QD: once daily; RR: relative risk; RRR: relative risk reduction; SC: subcutaneous; TIA: transient ischemic attack; TKA: total knee arthroplasty; VTE: venous thromboembolism. Confidence intervals (CIs) are reported at the level used in each original publication and are indicated for each estimate; the PACIFIC trials reported 90% CIs, whereas AZALEA-TIMI 71 reported 95% CIs. Estimates reported without a confidence interval in the source are presented descriptively. ROXI-VTE-I and ROXI-VTE-II report Bayesian posterior probabilities rather than confidence intervals.

**Table 3:** Design and outcomes of phase III trials of Factor XI/FXIa inhibitors.

Study Name	Medication Name	Type of Drug	Dose	Comparator	Condition of Interest	N	Status	Estimated Duration—Follow-Up	Results/Primary Endpoints	Secondary Endpoints
**OCEANIC-AF** (NCT05643573)	Asundexian	Small molecule	50 mg QD	Apixaban	Atrial Fibrillation	14,810	Terminated early (IDMC recommendation)	Median follow-up ~11.5 months	• 3.8-fold increase in Stroke or SE (1.3% vs. 0.4%) (HR 3.79; 95% CI 2.46–5.83) • 68% reduction in Major Bleeding (0.2% vs. 0.7%) (HR 0.32; 95% CI 0.18–0.55)	Net clinical benefit (composite stroke/SE/ISTH major bleeding)
**LIBREXIA-AF** (NCT05757869)	Milvexian	Small molecule	100 mg BID	Apixaban (5 mg or 2.5 mg BID per label)	Atrial Fibrillation/Atrial Flutter	~15,500 (estimated)	Ongoing	~4 years	• **Primary:** Stroke/SE (noninferiority) • **Safety: ** ISTH major bleeding/CRNMB	• Composite of stroke/SE/major bleeding (net clinical outcome) • All-cause death • Myocardial infarction
**LILAC-TIMI 76** (NCT05712200)	Abelacimab	Monoclonal antibody	150 mg SC monthly	Placebo	Atrial Fibrillation (unsuitable for standard anticoagulation); - Age ≥ 65 - CHA_2_DS_2_-VASc ≥ 3–4	~1900 (estimated)	Ongoing	~18 months (up to 30 months per participant)	• **Primary:** Stroke/SE (efficacy vs. placebo)	• **Safety:** Major bleeding
**OCEANIC-STROKE** (NCT05686070)	Asundexian	Small molecule	50 mg QD	Placebo (+antiplatelet therapy)	Noncardioembolic Ischemic Stroke/TIA	12,327	Completed	Median follow-up: 567 days (event-driven trial)	• 26% reduction in ischemic stroke (6.2% vs. 8.4%) (HR 0.74; 95% CI 0.65–0.84; *p* < 0.001) • No increase in major bleeding (1.9% vs. 1.7%) (HR 1.10; 95% CI 0.85–1.44; *p* = 0.46)	• **CV death/MI/stroke**: 9.2% vs. 11.1% (HR 0.83; 95% CI 0.74–0.92; *p* < 0.001) • **All-cause death/MI/stroke**: 10.5% vs. 12.3% (HR 0.85; 95% CI 0.77–0.95; *p* = 0.003) • **Disabling/fatal stroke**: 2.1% vs. 3.0% (HR 0.69; 95% CI 0.55–0.87)
**LIBREXIA-STROKE** (NCT05702034)	Milvexian	Small molecule	25 mg BID	Placebo (+antiplatelet therapy)	Noncardioembolic Ischemic Stroke/TIA	~15,000 (estimated)	Ongoing	Event-driven	• **Primary:** Ischemic stroke	• **Safety:** Major bleeding
**LIBREXIA-ACS** (NCT05754957)	Milvexian	Small molecule	25 mg BID	Placebo (+antiplatelet therapy)	Acute Coronary Syndrome	14,194	Terminated early (interim futility review)	Event-driven	• No reduction in CV death/MI/ischemic stroke (5.4% vs. 5.1%) (HR 1.05; 95% CI 0.91–1.21; *p* = 0.50) • No increase in intracranial/fatal bleeding, BARC 3c or 5 (0.3% vs. 0.3%) (HR 1.04; 95% CI 0.58–1.87; *p* = 0.88)	**• CV death/MI/ischemic stroke/MALE/symptomatic VTE:** 5.7% vs. 5.4% (HR 1.04; 95% CI 0.90–1.20) **• All-cause death/MI/ischemic stroke:** 6.1% vs. 5.7% (HR 1.05; 95% CI 0.92–1.21) **• All-cause death:** 2.2% vs. 2.3% (HR 0.96; 95% CI 0.77–1.20)
**ASTER ** (NCT05171049)	Abelacimab	Monoclonal antibody	150 mg SC monthly	Apixaban: - 10 mg BID for 7 days - 5 mg BID	Cancer-associated VTE	~1150	Terminated early (interim analysis)	—	• **Primary**: Recurrent VTE • **Safety**: Major/CRNMB	—
**MAGNOLIA** (NCT05171075)	Abelacimab	Monoclonal antibody	150 mg SC monthly	Dalteparin: - 200 IU/kg/day (×1 month) - 150 IU/kg/day	Cancer-Associated VTE (GI/GU Cancers)	~417	Terminated early	—	• **Primary**: Recurrent VTE • **Safety**: Major bleeding	—

Abbreviations: ACS: acute coronary syndrome; AF: atrial fibrillation; BARC: Bleeding Academic Research Consortium; BID: twice daily; CHA_2_DS_2_-VASc: congestive heart failure, hypertension, age ≥ 75 years (2 points), diabetes mellitus, stroke or transient ischemic attack (2 points), vascular disease, age 65–74 years, sex category (female); CI: confidence interval; CRNMB: clinically relevant non-major bleeding; CV: cardiovascular; GI: gastrointestinal; GU: genitourinary; HR: hazard ratio; IDMC: independent data monitoring committee; ISTH: International Society on Thrombosis and Haemostasis; IU: international units; MALE: major adverse limb events; MI: myocardial infarction; QD: once daily; SC: subcutaneous; SE: systemic embolism; TIA: transient ischemic attack; VTE: venous thromboembolism. All confidence intervals are 95% confidence intervals, as reported in the original phase III trial publications.

## Data Availability

No new data were created or analyzed in this study. Data sharing is not applicable to this article.
